# Long-term efficacy and safety of siponimod in patients with secondary progressive multiple sclerosis: Analysis of EXPAND core and extension data up to >5 years

**DOI:** 10.1177/13524585221083194

**Published:** 2022-04-05

**Authors:** Bruce AC Cree, Douglas L Arnold, Robert J Fox, Ralf Gold, Patrick Vermersch, Ralph HB Benedict, Amit Bar-Or, Daniela Piani-Meier, Nicolas Rouyrre, Shannon Ritter, Ajay Kilaru, Goeril Karlsson, Gavin Giovannoni, Ludwig Kappos

**Affiliations:** Department of Neurology, UCSF Weill Institute for Neurosciences, University of California San Francisco, San Francisco, CA, USA; NeuroRx Research, and Montreal Neurological Institute and Hospital, Department of Neurology and Neurosurgery, McGill University, Montreal, QC, Canada; Mellen Center for Treatment and Research in Multiple Sclerosis, Neurological Institute, Cleveland Clinic, Cleveland, OH, USA; Department of Neurology, St. Josef-Hospital and Ruhr-University Bochum, Bochum, Germany; Univ. Lille, INSERM U1172 LilNCog, CHU Lille, FHU Precise, Lille, France; Department of Neurology, University at Buffalo, Buffalo, NY, USA; Center for Neuroinflammation and Experimental Therapeutics and Department of Neurology, Perelman School of Medicine, University of Pennsylvania, Philadelphia, PA, USA; Novartis Pharma AG, Basel, Switzerland; Novartis Pharma AG, Basel, Switzerland; Novartis Pharma AG, Basel, Switzerland; Novartis Pharma AG, Basel, Switzerland; Novartis Pharma AG, Basel, Switzerland; Blizard Institute, Barts and The London School of Medicine and Dentistry, Queen Mary University of London, London, UK; Neurologic Clinic and Policlinic, Departments of Medicine, Clinical Research, Biomedicine and Biomedical Engineering, University Hospital, University of Basel, Basel, Switzerland

**Keywords:** Confirmed disability progression, confirmed cognitive worsening, cortical gray matter, secondary progressive multiple sclerosis, siponimod, S1P modulator

## Abstract

**Background:**

Siponimod significantly reduced the risk of confirmed disability progression (CDP), worsening in cognitive processing speed (CPS), relapses, and magnetic resonance imaging (MRI) measures of brain atrophy and inflammation versus placebo in secondary progressive multiple sclerosis (SPMS) patients in the Phase 3 EXPAND study.

**Objective:**

The aim of this study was to assess long-term efficacy and safety of siponimod 2 mg/day from the EXPAND study including the extension part, up to > 5 years.

**Methods:**

In the open-label extension part, participants receiving placebo during the core part were switched to siponimod (placebo-siponimod group) and those on siponimod continued the same treatment (continuous siponimod group).

**Results:**

Continuous siponimod reduced the risk of 6-month CDP by 22% (hazard ratio (HR) (95% confidence interval (CI)): 0.78 (0.66–0.92) *p* = 0.0026) and 6-month confirmed worsening in CPS by 23% (HR (95% CI): 0.77 (0.65–0.92) *p* = 0.0047) versus the placebo-siponimod group. Sustained efficacy on annualized relapse rate, total and regional brain atrophy, and inflammatory disease activity was also observed. No new, unexpected safety signals for siponimod were identified over the long term.

**Conclusion:**

The sustained efficacy and consistent long-term safety profile of siponimod up to > 5 years support its clinical utility for long-term treatment of SPMS. Benefits in the continuous siponimod versus placebo-siponimod group highlight the significance of earlier treatment initiation.

**Trial registration number:**

NCT01665144

## Introduction

Approximately 85% of patients with multiple sclerosis (MS) have a relapsing–remitting phenotype at the onset of disease, with ~20%–40% of these patients developing secondary progressive multiple sclerosis (SPMS) within 10 years, and up to ~50% within 20 years, after onset.^1–4^ SPMS is associated with reduced relapse activity (⩽ 30% of patients experience relapses after progression has started) and gradual worsening of disability and progressive neurological deterioration independent of relapses.^1,5–8^ The development of effective and safe therapies for SPMS has proved challenging. Although certain disease-modifying therapies evaluated in SPMS populations, including natalizumab,^9^ demonstrated beneficial effects on inflammatory activity, they failed to show consistent effects on disability progression.^9–12^ Siponimod is the first disease-modifying therapy that significantly reduced the risk of disability progression and decline in cognitive processing speed (CPS) in a large Phase 3 study in SPMS patients (EXPAND) in addition to reducing inflammatory disease activity.^13^ Results from the European interferon beta-1b^12^ and mitoxantrone^14^ studies showed reductions in time to confirmed disability progression (CDP), but the cohorts were not representative of a typical broad SPMS population. In comparison to other studies in SPMS,^9,10,13^ the interferon beta-1b European study assessed a younger, more inflammatory population with a higher percentage of patients with relapses in the 2 years prior to the study, a higher mean number of gadolinium-enhancing (Gd+) lesions at baseline and a placebo group showing a much higher on-study relapse rate. The mitoxantrone study was too small to provide confident estimates and, furthermore, included a mix of MS phenotypes.

Siponimod selectively modulates sphingosine 1-phosphate_1,5_ (S1P_1_,_5_) receptors,^3,15^ resulting in reduced lymphocyte egress from lymph nodes, thus eliciting an anti-inflammatory response and preventing recirculation of peripheral lymphocytes to the central nervous system (CNS).^16,17^ Siponimod penetrates the CNS and, based on evidence from preclinical studies, has the potential to exert direct beneficial effects on compartmentalized CNS inflammation, neurodegeneration, and remyelination.^18–23^

The ongoing extension part of the EXPAND study aims to assess the long-term efficacy and safety of siponimod and, as all participants in the extension are receiving siponimod, the effects of earlier versus later initiation of siponimod treatment in patients with SPMS.

## Patients and methods

### Study design and participant population

The core part of the Phase 3 EXPAND study was a multicenter, randomized (2:1), double-blind, parallel-group, placebo-controlled, variable-treatment-duration, event-driven study investigating the efficacy and safety of siponimod versus placebo in participants with SPMS (*N* = 1651), with a median study duration of 21 months (range: 0.2–37 months).^13^

**Figure 1.**
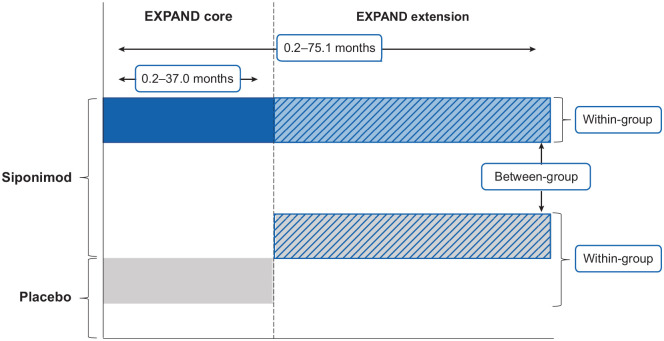
Comparison of short-term versus long-term treatment and early versus later treatment initiation with siponimod.

Given the event-driven design, duration in the core part before transitioning to the open-label extension part varied for individual participants and ranged from approximately 12 up to 37 months. Participants receiving siponimod 2 mg/day in the core part were maintained on siponimod (continuous siponimod group) and those receiving placebo also switched to open-label siponimod 2 mg/day (placebo-siponimod group) in the extension part (Figure 1). Original treatment assignment was not revealed until the core part was unblinded.

The cutoff date for the core + extension analyses was 6 April 2019, when the majority of patients had reached at least Month 36 of the extension part. With the variable duration of the core part, the mean/median duration among all patients included in the analyses was 45.1/53.1 months (range 0.2–75.1 months; Figure S1) at the time of the cutoff date. The protocol of the EXPAND study (NCT01665144) was approved by the relevant institutional review board or ethics committee at each trial site and all participants provided written informed consent.

### Study outcomes and assessments

Time to 6-month CDP was based on the Expanded Disability Status Scale (EDSS) score, assessed at core baseline and every 3 months in the core part, and for the first year of the extension part and every 6 months thereafter. Time to 6-month confirmed meaningful (⩾ 4 points) worsening in CPS was measured using the Symbol Digit Modalities Test (SDMT) assessed at baseline and every 6 months in the core and extension parts. Annualized relapse rate (ARR) was measured for confirmed relapses.

Magnetic resonance imaging (MRI) measures included total brain, cortical gray matter (cGM), and thalamic volume loss; T2 lesion volume change; and mean cumulative number of new/enlarging T2 lesions, which were assessed yearly during the core part, and after 1 year in the extension part and then biyearly thereafter for the rest of the extension part. Due to safety concerns regarding gadolinium exposure in the older patient population and the low degree of useful additional data beyond the number of new/enlarging T2 lesions, T1 Gd+ lesions were not measured in the extension part. Within-group analyses compared Months 0–12 of the core part and Months 0–12 of the extension part as well as the total core and extension parts. To account for variable exposure time, within-group comparison of the total core (median 21 months) and total extension parts (median 36 months) was performed using annualized changes in these MRI parameters. The percent change relative to the extension part baseline was derived accounting for the change during the core part. For between-group comparisons, change from core baseline to Month 36 in extension (up to 60 months total follow-up) was measured for the MRI endpoints. Safety analyses summarized the most common adverse events (AEs) and AEs of special interest in patients who received at least one dose of siponimod during the core or extension parts.

### Subgroup analyses

Outcomes for CDP and confirmed cognitive worsening (CCW) were assessed in the subgroup of participants with active SPMS (relapses in the 2 years prior to the core part screening and/or ⩾ 1 T1 Gd+ lesion at baseline in the core part)^13^ and SPMS without such activity (“non-active” SPMS; no relapses 2 years pre-study and no Gd+ lesions at baseline in the core part).^24^ MRI outcomes were also measured for both active and non-active SPMS subgroups and were reported elsewhere.^25^

### Statistical analysis

Comparative efficacy analyses were based on the intention-to-treat principle and compared the two treatment groups as per randomization in the combined full analysis set (all randomly assigned and treated patients (core + extension data)) from the core part. Time to 6-month CDP and time to 6-month CCW were analyzed using the Cox proportional hazards model with randomized treatment, country/region, baseline EDSS score (for CDP) or SDMT (for CCW), and SPMS group (with/without superimposed relapses; baseline definition) as covariates, using combined assessments for both CDP and CCW data from the core and extension parts. The between-group comparisons of ARR were analyzed using a negative binomial model. MRI data were analyzed using non-parametric methods and models for repeated measures; within-group participant covariance was modeled using a compound symmetry covariance matrix structure. Comparisons of percentage changes between the core and extension parts were made using the Wilcoxon signed-rank test. Safety was assessed in all participants from both the core and extension parts (combined safety set) for the treatment received. Descriptive statistics on the safety set were used to summarize AEs, AEs leading to discontinuation and serious adverse events (SAEs).

## Results

### Participant disposition and baseline characteristics

**Figure 2.**
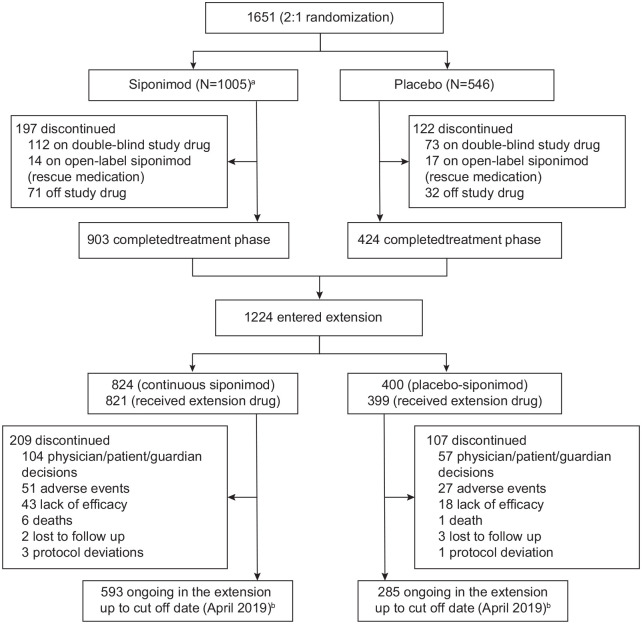
Participant disposition (overall population). Of the 1651 participants who were randomized (randomized set), 1646 received ⩾ 1 dose of randomized treatment (siponimod 2 mg or placebo) in the core part and were included in the analysis (full analysis set); 1224 participants entered the extension part; and 1220 received ⩾ 1 dose of open-label siponimod in the extension part. ^a^5 participants did not receive the study drug.^b^Participants not included: those with a disposition reason that was not available in the database or an adverse event that occurred after cutoff date (6 April 2019) for 19 participants in the continuous siponimod group and 7 in the placebo-siponimod group.

Of the 1651 participants randomized in the core part of the EXPAND study, a total of 1220 participants entered the extension part and received open-label siponimod (821 in the continuous siponimod group and 399 in the placebo-siponimod group; Figure 2). Further information on participant disposition in the core part was previously reported.^13^ At the time of the cutoff in April 2019, 316 (25.9%) patients who had entered the extension had discontinued, with 593 (72.2%) of the continuous siponimod group and 285 (71.4%) of the placebo-siponimod group continuing in the study and having completed Month 36 of the extension part. The active SPMS subgroup included 782 participants who were randomized in the core part, and of these, 582 participated in the extension. The non-active SPMS subgroup included 830 participants who were randomized in the core part, and of these, 612 participated in the extension. Demographic and disease characteristics of participants at baseline in both the core and extension parts were balanced (Table 1).

**Table 1.** Patient demographics and baseline disease characteristics.

Parameter	Core study (randomized set),^23^ *N* = 1641		Participants entering extension part, *N* = 1224	
	Siponimod (*N* = 1105)	Placebo (*N* = 546)	Siponimod (*N* = 824)	Placebo (*N* = 400)
Age (years)	48.0 ± 7.8	48.1 ± 7.9	47.8 ± 7.8	48.5 ± 8.1
>41 years, *n* (%)	917 (83.0)	443 (81.1)	678 (82.3)	324 (81.1)
Time since onset of MS symptoms (years)	17.1 ± 8.4	16.2 ± 8.2	16.9 ± 8.3	16.2 ± 8.4
Time since conversion to SPMS (years)	3.9 ± 3.6	3.6 ± 3.3	3.7 ± 3.5	3.5 ± 3.2
Time since onset of the last relapse (years)	5.15 ± 5.13	4.52 ± 4.61	4.99 ± 5.04	4.82 ± 4.84
Absence of relapses in the last 2 years prior to screening, *n* (%)^a^	712 (64)	343 (63)	521 (63)	260 (65)
Absence of relapses in the last year prior to screening, *n* (%)^a^	878 (79)	416 (76)	651 (79)	311 (78)
EDSS score	5.4 ± 1.1	5.4 ± 1.0	5.4 ± 1.1	5.4 ± 1.0
Median (range)	6.0 (2.0–7.0)	6.0 (2.5–7.0)	6.0 (2.5–7.0)	6.0 (2.5–7.0)
SDMT score	38.9 ± 13.99	39.6 ± 13.34	38.8 ± 14.09	40.6 ± 13.11
Median (range)	40.0 (0–83)	42.0 (0–81)	40.0 (0–80)	43.0 (1–81)
Absence of Gd+ T1 lesions at baseline, *n* (%)^a^	833 (75)	415 (76)	613 (74)	312 (78)
Total volume of lesions on T2-weighted images (mm^3^)	15,632 ± 16,268	14,694 ± 15,620	15,165 ± 15,760	13,702 ± 15,106
Normalized brain volume (cm^3^)	1422 ± 86	1425 ± 88	1423 ± 87	1423 ± 87

MS: multiple sclerosis; SPMS: secondary progressive multiple sclerosis; EDSS: Expanded Disability Status Scale; SDMT: Symbol Digit Modalities Test; Gd+: gadolinium-enhancing.

All randomized set. Data represented as mean ± SD, unless otherwise specified.

a The numbers and percentages of participants with missing screening or baseline observations are not displayed.

### 6-month CDP

#### Overall study population

**Figure 3.**
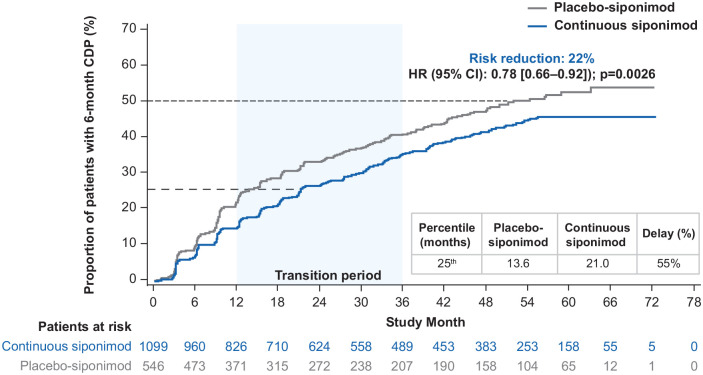
Kaplan–Meier estimate of the time to 6-month confirmed disability progression in the combined core and extension parts (combined FAS—overall population). Combined FAS includes all available EDSS data from the start of the core part to the cutoff date of the extension part. Subjects without baseline EDSS assessment were excluded from the analysis. CDP: confirmed disability progression; CI: confidence interval; EDSS: Expanded Disability Status Scale; FAS: full analysis set; HR: hazard ratio.

Risk of 6-month CDP on EDSS was significantly reduced by 22% in participants receiving continuous siponimod versus those in the placebo-siponimod group (hazard ratio (HR) 95% confidence interval (CI): 0.78 (0.66–0.92) *p* = 0.0026; Figure 3). The median time to 6-month CDP was not reached in the continuous siponimod group and was 51.7 months for the placebo-siponimod group. There was a 55% delay in 6-month CDP for the 25th percentile in the continuous siponimod group versus the placebo-siponimod group. For interim percentiles, see Table S1.

#### Subgroups of participants with active and non-active SPMS

**Figure 4.**
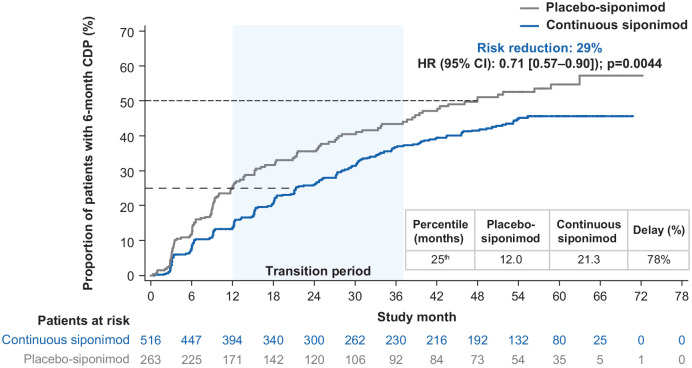
Kaplan–Meier estimate of the time to 6-month confirmed disability progression in the combined core and extension parts (combined FAS—active SPMS). Combined FAS includes all available EDSS data from the start of the core part to the cutoff date of the extension part. Subjects without baseline EDSS assessment were excluded from the analysis. CDP: confirmed disability progression; CI: confidence interval; EDSS: Expanded Disability Status Scale; FAS: full analysis set; HR: hazard ratio; SPMS: secondary progressive multiple sclerosis.

In participants with active SPMS, the risk of 6-month CDP was reduced by 29% (HR (95% CI): 0.71 (0.57‒0.90) *p* = 0.0044) for the continuous siponimod group versus the placebo-siponimod group (Figure 4). The median time to 6-month CDP was not reached for the continuous siponimod group and was 48.0 months for the placebo-siponimod group. A delay of > 75% in 6-month CDP at the 25th percentile was recorded in the continuous siponimod group versus the placebo-siponimod group. For interim percentiles, see Table S2. In participants with non-active SPMS, the risk of 6-month CDP was numerically, but not significantly, reduced by 12.5% (HR (95% CI): 0.88 (0.69–1.11)) and time to 6-month CDP for the 25th percentile was prolonged by ~36% in the continuous siponimod group versus the placebo-siponimod group (21.0 vs 15.4 months). Time to progression in the placebo-siponimod group was 28% longer in participants with non-active SPMS than active SPMS (15.4 vs 12.0 months at the 25th percentile; percentiles are presented in Table S3).

### 6-monthCCW

#### Overall study population

**Figure 5.**
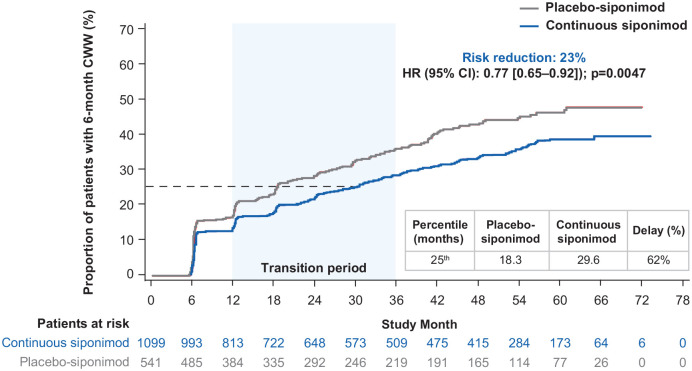
Kaplan–Meier estimate of the time to 6-month confirmed clinically meaningful worsening in CPS in the combined core and extension parts (combined FAS—overall population). Combined FAS includes all available SDMT data from the start of the core part to the cutoff date of the extension part. Subjects without baseline SDMT assessment were excluded from the analysis. CCW: confirmed cognitive worsening; CI: confidence interval; CPS: cognitive processing speed; FAS: full analysis set; HR: hazard ratio; SDMT: Symbol Digit Modalities Test.

The risk of 6-month confirmed worsening in CPS was reduced by 23% (HR (95% CI): 0.77 (0.65–0.92) *p* = 0.0047) in the continuous siponimod group versus the placebo-siponimod group (Figure 5). The median was not reached for CCW in the continuous siponimod or placebo-siponimod groups, while there was a > 60% delay in 6-month CCW for the 25th percentile in the continuous siponimod group versus the placebo-siponimod group. For interim percentiles, see Table S3.

#### Subgroups of participants with active and non-active SPMS

**Figure 6.**
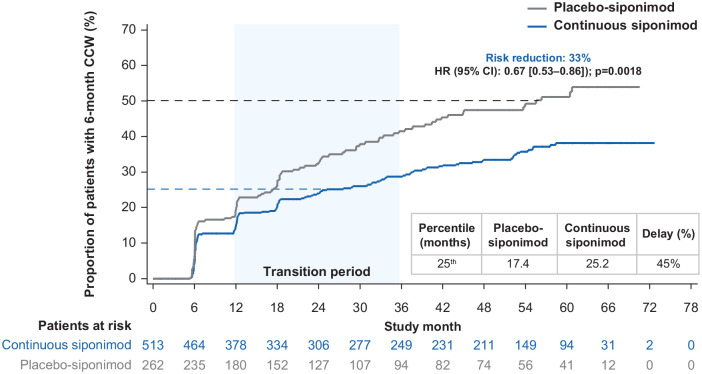
Kaplan–Meier estimate of the time to 6-month confirmed clinically meaningful worsening in CPS in the combined core and extension parts (combined FAS—active SPMS). Combined FAS includes all available SDMT data from the start of the core part to the cutoff date of the extension part. Subjects without baseline SDMT assessment were excluded from the analysis. CCW: confirmed cognitive worsening; CI: confidence interval; CPS: cognitive processing speed; FAS: full analysis set; HR: hazard ratio; SDMT: Symbol Digit Modalities Test; SPMS: secondary progressive multiple sclerosis.

In participants with active SPMS, the risk of 6-month CCW for the continuous versus placebo-siponimod groups was reduced by 33% (HR (95% CI): 0.67 (0.53‒0.86) *p* = 0.0018; Figure 6). The median time to 6-month CCW in the placebo-siponimod group was 55.5 months and was not reached in the continuous siponimod group. A 45% delay in 6-month CCW was recorded at the 25th percentile in the continuous siponimod group versus the placebo-siponimod group. For interim percentiles, see Table S4. In non-active SPMS participants, the risk of 6-month CCW was numerically, but not significantly, reduced by 12.3% (HR (95% CI): 0.88 (0.68–1.14)) and time to 6-month CCW for the 25th percentile was prolonged by 17% (30.4 vs 26.0 months), favoring the continuous siponimod group. The time to cognitive worsening in the placebo-siponimod group was 49% longer in participants with non-active SPMS than active SPMS (26.0 vs 17.4 months at the 25th percentile; percentiles are presented in Table S4).

### ARR

#### Between-group comparison

**Figure 7.**
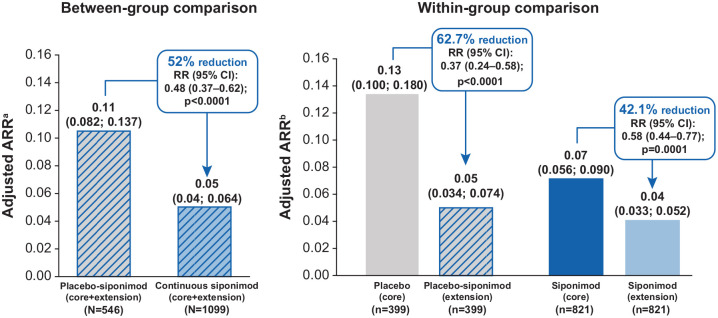
Adjusted ARR for between-group and within-group comparisons (overall population). ARR: annualized relapse rate; CI: confidence interval; EDSS: Expanded Disability Status Scale; Gd+: gadolinium-enhancing; RR: rate ratio; SPMS: secondary progressive multiple sclerosis. ^a^Negative binomial regression model adjusted for the core part treatment group. ^b^Poisson regression model adjusted for the treatment period (core part and extension part). Both models were also adjusted for country, baseline EDSS score, SPMS group (with/without superimposed relapses; baseline definition), and baseline number of T1 Gd+ lesions categories.

In the combined core and extension parts, there was a statistically significant reduction in ARR (52.0%, *p* < 0.0001) in the continuous siponimod group versus the placebo-siponimod group (Figure 7).

#### Within-group comparison

The ARR during the core versus extension parts for participants originally assigned to placebo showed an expected decrease from 0.13 to 0.05 following switch to siponimod—a reduction of 62.7% (rate ratio (RR) (95% CI): 0.37 (0.24–0.58) *p* < 0.0001). For participants originally assigned to siponimod, the ARR further decreased from 0.07 in the core part to 0.04 in the extension part—a reduction of 42.1% (RR (95% CI): 0.58 (0.44–0.77) *p* = 0.0001).

### MRI

#### Between-group comparisons

**Figure 8.**
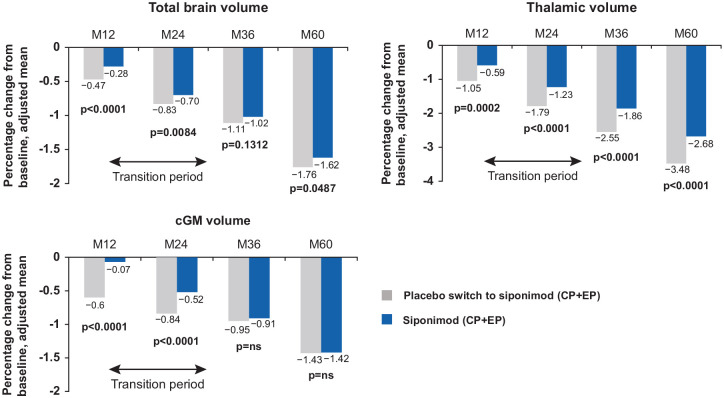
Mean cumulative percentage change from baseline during the study in total brain, thalamic, and cGM volume (between-group comparison—overall population). Total brain volume: placebo switch to siponimod (*N* = 457); siponimod (*N* = 929). Thalamic volume: placebo switch to siponimod (*N* = 451); siponimod (*N* = 920). cGM volume: placebo switch to siponimod (*N* = 448); siponimod (*N* = 920). MMRM model: percentage change from baseline adjusted for visit, treatment, age, number of Gd+ T1 lesions at baseline, T2 lesion volume (mm^3^) at baseline, superimposed relapses at baseline, visit by treatment interaction (and baseline normalized brain tissue volume, where applicable). cGM: cortical gray matter; CP: core part; EP: extension part; Gd+: gadolinium-enhancing; M: month; MMRM: mixed model repeated measures; ns: not significant.

**Figure 9.**
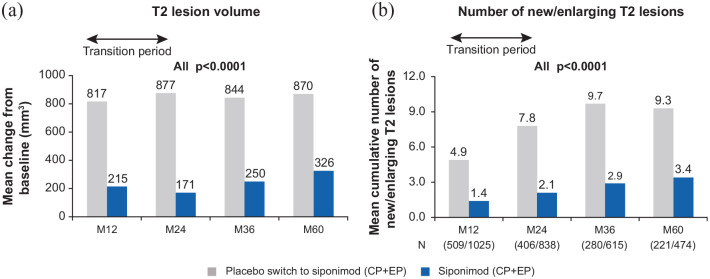
Mean change from baseline during the study in T2 lesion volume and the cumulative number of new/enlarging T2 lesions (between-group comparison—overall population). (a) Placebo switch to siponimod (*N* = 496); siponimod (*N* = 999). MMRM model: percentage change from baseline adjusted for visit, treatment, age, number of Gd+ T1 lesions at baseline, T2 lesion volume (mm^3^) at baseline, superimposed relapses at baseline, visit by treatment interaction (and baseline normalized brain tissue volume, where applicable) and (b) *p*-values from the Wilcoxon signed-rank test for the differences between treatment groups. CP: core part; EP: extension part; Gd+: gadolinium-enhancing; M: month.

After 60 months of follow-up, the extent of total brain volume loss (cumulative percentage change from baseline: −1.62% vs −1.76%; *p* < 0.05) and of thalamic volume loss (cumulative percentage change from baseline: −2.68% vs −3.48%; *p* < 0.0001) was reduced in the continuous siponimod versus the placebo-siponimod group. Switch to siponimod from placebo on entry to the extension reduced cGM volume loss to such an extent that there was no longer a significant between-group difference in cumulative cGM volume loss at Month 60. (−1.42% vs −1.43%; Figure 8). T2 lesion volume change from baseline and cumulative number of new/enlarging T2 lesions were also significantly reduced in the continuous siponimod versus placebo-siponimod group after 60 months of follow-up (326 vs 870 mm^3^ and 3.4 vs 9.3, respectively; both *p*s < 0.0001; Figure 9). The mean number of new/enlarging T2 lesions from the previous visit was similar between placebo-siponimod and continuous siponimod groups after the transition period (M60 with reference to M36; Figure S2).

#### Within-group comparisons

**Figure 10.**
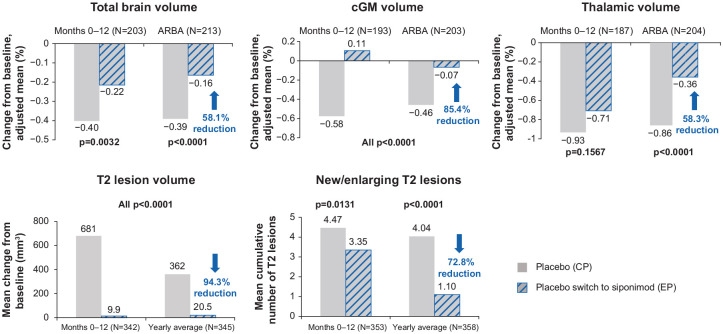
Within-group comparison: MRI outcomes in the placebo-siponimod switch group—overall population. At each time point, only participants with a value both at the core part visit and the corresponding extension part visit are included; percentage change relative to the start of the extension part was derived by accounting for the change during the core part; *p*-values from the Wilcoxon signed-rank test comparing percentage changes between the core part and the extension part within each group. ARBA is derived from percent change to last visit during the core part (median 21 months) and during the extension part (median 36 months). ARBA: annualized rate of brain atrophy; cGM: cortical gray matter; CP: core part; EP: extension part; M: month.

Over the entire extension period, the placebo-siponimod group showed pronounced reductions in the annualized rate of brain atrophy (ARBA) for total brain (58.1%), cGM (85.4%), and thalamus (58.3%; all *p*s < 0.0001; Figure 10). The yearly T2 lesion volume change and cumulative number of new/enlarging T2 counts were reduced by 94.3% and 72.8%, respectively, in this group (*p* < 0.0001; Figure 10). Complete suppression of cGM atrophy and no increase in T2 lesion volume were observed within the first 12 months on switching from placebo to siponimod (M0–12 of the extension).

**Figure 11.**
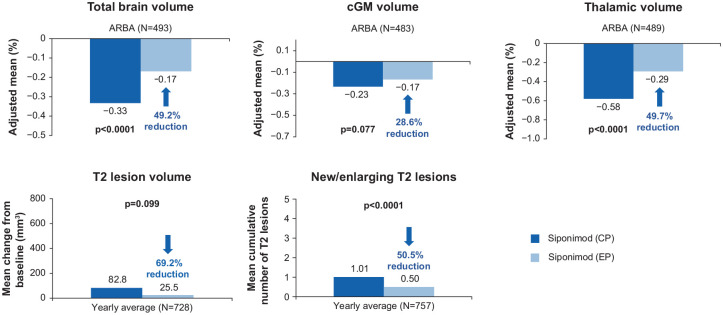
Within-group comparison: MRI outcomes in the continuous siponimod group—overall population. At each time point, only participants with a value both at the core part visit and the corresponding extension part visit are included; the yearly average is derived by standardizing the change to the last visit to 360 days; *p*-values from the Wilcoxon signed-rank test comparing percentage changes between the core part and the extension part within each group. ARBA is derived from percent change to last visit during the core part (median 21 months) and during the extension part (median 36 months). CP: core part; EP: extension part; M: month.

In the continuous siponimod group, there was a further reduction in ARBA, suggesting a continuing accrual of efficacy on brain atrophy, a further reduction in the formation of new/enlarging T2 lesions, and an almost complete suppression of any increase in T2 lesion volume (Figure 11).

### Safety analysis

**Figure 12.**
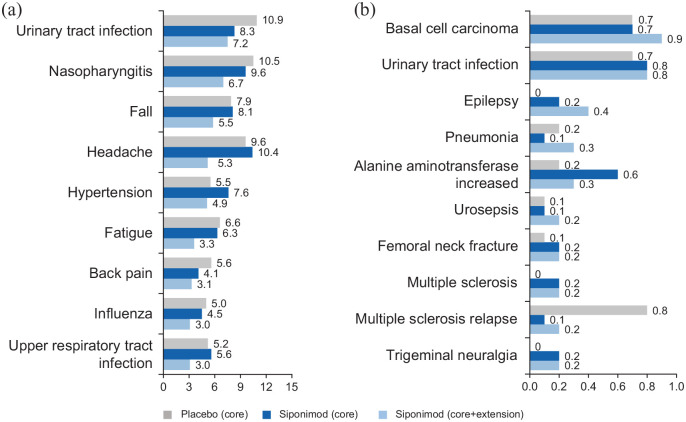
IRs for AEs and SAEs per 100 PY (safety set—overall population). IRs for (a) AEs (reported with IR of at least 3.0 for siponimod) and (b) SAEs (reported with IR of at least 0.1 for siponimod) per 100 PY. SAEs were reported by investigator if MS/MS relapse was unusually severe or medically unexpected as per protocol. IRs were computed as the number of participants with an AE divided by the total exposure for the AE (i.e. cumulative exposure until the first occurrence or 5-year cutoff date (6 April 2019)). AE: adverse event; IR: incidence rate; PY: patient-years; SAE: serious adverse event.

The most frequently observed AEs in the long-term siponimod dataset were consistent with those observed in the core part, with no increase in exposure-adjusted incidence rates (IRs; per 100 patient-years (PY)) during exposure to siponimod treatment (⩾ 36 months; Figure 12(a)). The pattern of most frequent SAEs experienced by 463 participants (30.5%; IR = 10.9 per 100 PY) was also consistent with the core part (Figure 12(b)). This pattern was also true for AEs leading to siponimod discontinuation (179 participants (11.8%; IR = 3.6 per 100 PY)). The causes of the 16 deaths that occurred in participants treated with siponimod (4 in the core and 12 in the extension) since the start of the core part up to > 5 years (Table 2) were heterogeneous with no signal for a particular organ system. In the active SPMS groups, the AE profile up to > 5 years was in line with that of the overall population (Figure S3).

**Table 2.** Summary of AEs, SAEs, and deaths (safety set—overall population).

Event	Core (Placebo) *N* = 546 *n* (%); IR (95% CI)	Core (Siponimod) *N* = 1099 *n* (%); IR (95% CI)	Core + extension (Siponimod) *N* = 1571 *n* (%); IR (95% CI)
AEs	446 (81.7); 172.9 (157.2–189.7)	981 (89.3); 249.2 (233.8–265.3)	1420 (93.6); 184.2 (174.7–194.0)
SAEs	74 (13.6); 9.5 (7.5–12.0)	189 (17.2); 12.1 (10.4–14.0)	463 (30.5); 10.9 (9.9–12.0)
AEs leading to study drug discontinuation	28 (5.1%); 3.4 (2.2–4.9)	86 (7.8%); 5.0 (4.0–6.2)	179 (11.8%); 3.6 (3.1–4.1)
Death^a^	4 (1%)	4 (<1%)	16 (1%)

AE: adverse event; SAE: serious adverse event; N: number of patients included in the analyses; n: number of patients with an adverse event; IR: incidence rate; CI: confidence interval.

Incidence rates were computed as the number of participants with an AE divided by the total exposure to the AE (i.e. cumulative exposure until the first occurrence or until the end of the extension).

a IRs not calculated.

Overall, there was no unexpected increase in the exposure-adjusted IRs of AEs of special interest with siponimod from the core part to the 5-year treatment period (Table S5). In particular, malignancies, based on risk search terms defined by standardized MedDRA queries or unspecified tumors, were reported in 5.1% of participants treated with siponimod (78 participants; IR = 1.6 per 100 PY (95% CI: 1.2–2.0)) over the long term versus 1.9% (21 participants; IR = 1.2 per 100 PY (95% CI: 0.8–1.9)) treated with siponimod in the core part. An increase in the IR of basal cell carcinoma was observed with longer term exposure in the extension, but other AEs of special interest, including bradyarrhythmia at treatment initiation, hypertension, and varicella-zoster virus (VZV), were in line with the core part (Figure 12). In addition, there were no cases of progressive multifocal leukoencephalopathy (PML), but one case of cryptococcal meningitis (CM) reported in the extension part. No new safety signals that are unexpected for siponimod or S1P modulators were identified with long-term treatment up to > 5 years.

## Discussion

The analyses of the combined EXPAND core and extension parts demonstrate consistent and sustained efficacy of siponimod up to > 5 years (range: 0.2–75.1 months) on all clinical and MRI outcomes assessed. These analyses underscore the benefit of earlier initiation of siponimod, while the safety profile of siponimod with treatment up to > 5 years was largely consistent with the core part^13^ and in line with other S1P receptor modulators.

In the continuous siponimod group, a delay in 6-month CDP on EDSS of 55% (overall SPMS population) and > 75% (active SPMS subgroup) at the 25th percentile, with corresponding risk reductions of 22% and 29% versus the placebo-siponimod group, reflects the sustained benefit of long-term siponimod treatment. A similar persistent treatment effect was observed for 6-month confirmed clinically meaningful worsening in CPS for the continuous siponimod group with delays of > 60% (overall SPMS population) and 45% (active SPMS subgroup), corresponding to risk reductions of 23% and 33% versus the placebo-siponimod group.

Although it has been observed that immunomodulatory therapies are less effective in non-active forms of MS, a recent assessment of the EXPAND core population indicated the potential usefulness of siponimod in treating SPMS with or without on-study relapses.^8^ The present analyses indicate that with longer observation periods, there is a numerical difference between the effect of continuous siponimod on time to 6-month CDP and worsening in CPS versus placebo-siponimod in non-active SPMS, despite switch to active treatment in the siponimod-placebo group. While remaining non-significant, this numerical difference is in line with that observed in the overall extension population. Participants with non-active SPMS appear to progress more slowly than those with active SPMS as suggested by the approximate 30%–50% longer time needed for a 6-month confirmed progression on either EDSS (25th percentile 15.4 vs 12.0 months) or SDMT (26.0 vs 17.4 months) in the respective placebo-siponimod arm. Therefore, a longer follow-up period may be needed to see the full treatment effect versus placebo in non-active SPMS. This could explain why a beneficial effect on clinical outcomes in the non-active SPMS patient population was not obvious during the core study,^8^ although a benefit was seen on the more sensitive and objective MRI measures. Significant effects of siponimod versus placebo were observed in the core study in both active and non-active SPMS patients for MRI measures related to neurodegeneration, including GM atrophy and magnetization transfer imaging.^25^

Adjusted ARR in the continuous siponimod group remained low over 5 years (0.04), showing sustained benefit with continued siponimod treatment. Adjusted ARR was also reduced after switch from placebo to siponimod (0.05) in the extension part. For the MRI outcomes, the between-group analyses comparing the continuous siponimod and placebo-siponimod groups from baseline up to > 5 years show that the placebo-siponimod group carried forward an increased burden of focal injury and brain volume loss. Persistent differences between the continuous siponimod and placebo-siponimod groups in measures of brain tissue integrity are in line with the clinical findings and further emphasize the importance of earlier treatment initiation.

For the within-group comparison of the extension and core parts, the placebo-siponimod switch group recapitulated pronounced reductions during the extension part after the switch to siponimod in total brain volume, cGM volume, and thalamic volume loss (58%–85% reduction), and inflammatory MRI lesion activity and T2 lesion volume change (73%–94% reduction). It is noteworthy that siponimod showed a complete suppression of average cGM volume loss and T2 lesion volume accumulation within the first 12 months of switching from placebo to siponimod in the extension part. While suppression of white matter inflammatory lesion activity by siponimod is clearly evident throughout the core and extension parts of EXPAND, the data also suggest a treatment effect on cGM that is greater than that expected from the suppression of white matter inflammation alone. This suggests that siponimod contributes an additional effect on cGM tissue integrity, beyond a reduction in peripherally-driven inflammation. Interestingly, in line with this notion, preclinical studies have shown remyelination and neuroprotective direct effects of siponimod, also independent of inflammation.^19,21,26^ Furthermore, as shown in subgroup analyses of the EXPAND core study in patients with active and non-active SPMS, beneficial effects of siponimod have been observed on reducing GM atrophy and decrease in MTR that were independent of pre-study relapses and baseline MRI activity.^25^ In the continuous siponimod group, beneficial effects on global, thalamic, and cortical brain volumes, and T2 lesion volume accrual and new/enlarging T2 lesion activity, increased even further during the extension period, in keeping with not only sustained but also increasing efficacy on those outcomes over the long term.

Several studies support a dual mechanism of action for siponimod acting both peripherally and centrally to target inflammation and neurodegeneration/myelination. The long-term efficacy observed in participants with SPMS across different clinical measures (including physical and cognitive disability, relapse, and MRI measures related to both inflammatory disease activity and neurodegeneration) seems to be consistent with this dual mechanism of action.^18–22,27^

Treatment with siponimod was generally well tolerated, even with the relatively older and more disabled population assessed in this study compared with the studies of other S1P receptor modulators in patients with relapsing MS.^28–30^ IRs of the most common AEs and SAEs were consistent with the core part. AEs of special interest, including bradyarrhythmia at treatment initiation, hypertension, and VZV, were also in line with the core part and other S1P modulators, particularly fingolimod.^31^ There was one case of CM in the extension part up to the data cutoff and no reported cases of PML. Cases of basal cell carcinoma were reported with long-term siponimod, and this is also in line with fingolimod^32^ and not unexpected. Causes of death were heterogeneous with no signal for a particular organ system.

In conclusion, the sustained clinical efficacy and consistent safety profile support the clinical utility of siponimod for the long-term treatment of SPMS. Persistent treatment differences on both clinical and MRI measures favoring participants on continuous siponimod treatment over those who switched from placebo to siponimod highlight the significance of earlier treatment initiation.

## Supplemental Material

sj-docx-1-msj-10.1177_13524585221083194 – Supplemental material for Long-term efficacy and safety of siponimod in patients with secondary progressive multiple sclerosis: Analysis of EXPAND core and extension data up to >5 years Click here for additional data file. Supplemental material, sj-docx-1-msj-10.1177_13524585221083194 for Long-term efficacy and safety of siponimod in patients with secondary progressive multiple sclerosis: Analysis of EXPAND core and extension data up to >5 years by Bruce AC Cree, Douglas L Arnold, Robert J Fox, Ralf Gold, Patrick Vermersch, Ralph HB Benedict, Amit Bar-Or, Daniela Piani-Meier, Nicolas Rouyrre, Shannon Ritter, Ajay Kilaru, Goeril Karlsson, Gavin Giovannoni and Ludwig Kappos in Multiple Sclerosis Journal

sj-docx-2-msj-10.1177_13524585221083194 – Supplemental material for Long-term efficacy and safety of siponimod in patients with secondary progressive multiple sclerosis: Analysis of EXPAND core and extension data up to >5 years Click here for additional data file. Supplemental material, sj-docx-2-msj-10.1177_13524585221083194 for Long-term efficacy and safety of siponimod in patients with secondary progressive multiple sclerosis: Analysis of EXPAND core and extension data up to >5 years by Bruce AC Cree, Douglas L Arnold, Robert J Fox, Ralf Gold, Patrick Vermersch, Ralph HB Benedict, Amit Bar-Or, Daniela Piani-Meier, Nicolas Rouyrre, Shannon Ritter, Ajay Kilaru, Goeril Karlsson, Gavin Giovannoni and Ludwig Kappos in Multiple Sclerosis Journal

sj-docx-3-msj-10.1177_13524585221083194 – Supplemental material for Long-term efficacy and safety of siponimod in patients with secondary progressive multiple sclerosis: Analysis of EXPAND core and extension data up to >5 years Click here for additional data file. Supplemental material, sj-docx-3-msj-10.1177_13524585221083194 for Long-term efficacy and safety of siponimod in patients with secondary progressive multiple sclerosis: Analysis of EXPAND core and extension data up to >5 years by Bruce AC Cree, Douglas L Arnold, Robert J Fox, Ralf Gold, Patrick Vermersch, Ralph HB Benedict, Amit Bar-Or, Daniela Piani-Meier, Nicolas Rouyrre, Shannon Ritter, Ajay Kilaru, Goeril Karlsson, Gavin Giovannoni and Ludwig Kappos in Multiple Sclerosis Journal

sj-docx-4-msj-10.1177_13524585221083194 – Supplemental material for Long-term efficacy and safety of siponimod in patients with secondary progressive multiple sclerosis: Analysis of EXPAND core and extension data up to >5 years Click here for additional data file. Supplemental material, sj-docx-4-msj-10.1177_13524585221083194 for Long-term efficacy and safety of siponimod in patients with secondary progressive multiple sclerosis: Analysis of EXPAND core and extension data up to >5 years by Bruce AC Cree, Douglas L Arnold, Robert J Fox, Ralf Gold, Patrick Vermersch, Ralph HB Benedict, Amit Bar-Or, Daniela Piani-Meier, Nicolas Rouyrre, Shannon Ritter, Ajay Kilaru, Goeril Karlsson, Gavin Giovannoni and Ludwig Kappos in Multiple Sclerosis Journal

sj-docx-5-msj-10.1177_13524585221083194 – Supplemental material for Long-term efficacy and safety of siponimod in patients with secondary progressive multiple sclerosis: Analysis of EXPAND core and extension data up to >5 years Click here for additional data file. Supplemental material, sj-docx-5-msj-10.1177_13524585221083194 for Long-term efficacy and safety of siponimod in patients with secondary progressive multiple sclerosis: Analysis of EXPAND core and extension data up to >5 years by Bruce AC Cree, Douglas L Arnold, Robert J Fox, Ralf Gold, Patrick Vermersch, Ralph HB Benedict, Amit Bar-Or, Daniela Piani-Meier, Nicolas Rouyrre, Shannon Ritter, Ajay Kilaru, Goeril Karlsson, Gavin Giovannoni and Ludwig Kappos in Multiple Sclerosis Journal

sj-docx-6-msj-10.1177_13524585221083194 – Supplemental material for Long-term efficacy and safety of siponimod in patients with secondary progressive multiple sclerosis: Analysis of EXPAND core and extension data up to >5 years Click here for additional data file. Supplemental material, sj-docx-6-msj-10.1177_13524585221083194 for Long-term efficacy and safety of siponimod in patients with secondary progressive multiple sclerosis: Analysis of EXPAND core and extension data up to >5 years by Bruce AC Cree, Douglas L Arnold, Robert J Fox, Ralf Gold, Patrick Vermersch, Ralph HB Benedict, Amit Bar-Or, Daniela Piani-Meier, Nicolas Rouyrre, Shannon Ritter, Ajay Kilaru, Goeril Karlsson, Gavin Giovannoni and Ludwig Kappos in Multiple Sclerosis Journal

sj-docx-7-msj-10.1177_13524585221083194 – Supplemental material for Long-term efficacy and safety of siponimod in patients with secondary progressive multiple sclerosis: Analysis of EXPAND core and extension data up to >5 years Click here for additional data file. Supplemental material, sj-docx-7-msj-10.1177_13524585221083194 for Long-term efficacy and safety of siponimod in patients with secondary progressive multiple sclerosis: Analysis of EXPAND core and extension data up to >5 years by Bruce AC Cree, Douglas L Arnold, Robert J Fox, Ralf Gold, Patrick Vermersch, Ralph HB Benedict, Amit Bar-Or, Daniela Piani-Meier, Nicolas Rouyrre, Shannon Ritter, Ajay Kilaru, Goeril Karlsson, Gavin Giovannoni and Ludwig Kappos in Multiple Sclerosis Journal

sj-docx-8-msj-10.1177_13524585221083194 – Supplemental material for Long-term efficacy and safety of siponimod in patients with secondary progressive multiple sclerosis: Analysis of EXPAND core and extension data up to >5 years Click here for additional data file. Supplemental material, sj-docx-8-msj-10.1177_13524585221083194 for Long-term efficacy and safety of siponimod in patients with secondary progressive multiple sclerosis: Analysis of EXPAND core and extension data up to >5 years by Bruce AC Cree, Douglas L Arnold, Robert J Fox, Ralf Gold, Patrick Vermersch, Ralph HB Benedict, Amit Bar-Or, Daniela Piani-Meier, Nicolas Rouyrre, Shannon Ritter, Ajay Kilaru, Goeril Karlsson, Gavin Giovannoni and Ludwig Kappos in Multiple Sclerosis Journal

sj-pdf-9-msj-10.1177_13524585221083194 – Supplemental material for Long-term efficacy and safety of siponimod in patients with secondary progressive multiple sclerosis: Analysis of EXPAND core and extension data up to >5 years Click here for additional data file. Supplemental material, sj-pdf-9-msj-10.1177_13524585221083194 for Long-term efficacy and safety of siponimod in patients with secondary progressive multiple sclerosis: Analysis of EXPAND core and extension data up to >5 years by Bruce AC Cree, Douglas L Arnold, Robert J Fox, Ralf Gold, Patrick Vermersch, Ralph HB Benedict, Amit Bar-Or, Daniela Piani-Meier, Nicolas Rouyrre, Shannon Ritter, Ajay Kilaru, Goeril Karlsson, Gavin Giovannoni and Ludwig Kappos in Multiple Sclerosis Journal
