# Supplementary material for: Long-term efficacy and safety of siponimod in patients with secondary progressive multiple sclerosis: Analysis of EXPAND core and extension data up to >5 years
Source: Mult Scler. 2022 Apr 5;28(10):1591–605. doi: 10.1177/13524585221083194 (PMC9315196; doi:10.1177/13524585221083194)
Supplement: sj-docx-1-msj-10.1177_13524585221083194 – Supplemental material for Long-term efficacy and safety of siponimod in patients with secondary progressive multiple sclerosis: Analysis of EXPAND core and extension data up to >5 years [file sj-docx-1-msj-10.1177_13524585221083194.docx]

**Figure S1. Core and extension study design**


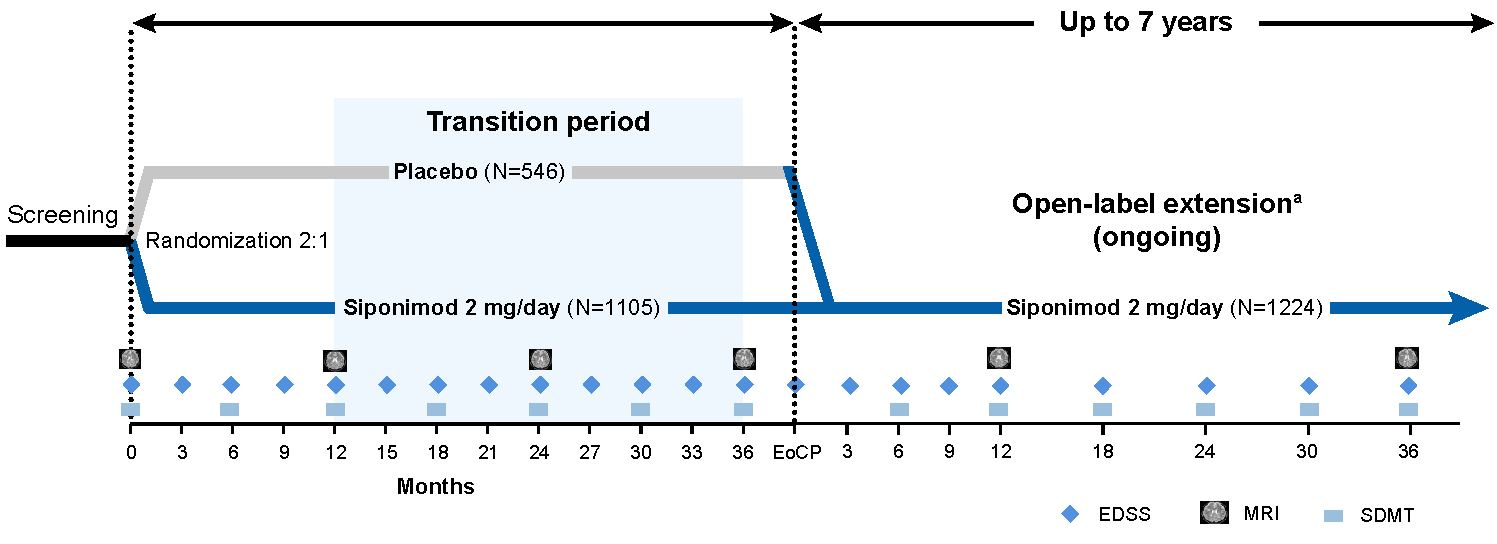


Extension data cutoff: April 2019 (Month 36 visit of extension); total study duration (core+extension): up to >5 years. ^a^Open-label treatment starts when the participant has a clinical event.

EDSS, Expanded Disability Status Scale; EoCP, end of core part; MRI, magnetic resonance imaging; SDMT, Symbol Digit Modalities Test.
