# Supplementary material for: Long-term efficacy and safety of siponimod in patients with secondary progressive multiple sclerosis: Analysis of EXPAND core and extension data up to >5 years
Source: Mult Scler. 2022 Apr 5;28(10):1591–605. doi: 10.1177/13524585221083194 (PMC9315196; doi:10.1177/13524585221083194)
Supplement: sj-docx-2-msj-10.1177_13524585221083194 – Supplemental material for Long-term efficacy and safety of siponimod in patients with secondary progressive multiple sclerosis: Analysis of EXPAND core and extension data up to >5 years [file sj-docx-2-msj-10.1177_13524585221083194.docx]

**Figure S2. Mean cumulative number of new/enlarging T2 lesions from the previous scan (overall population)**

**
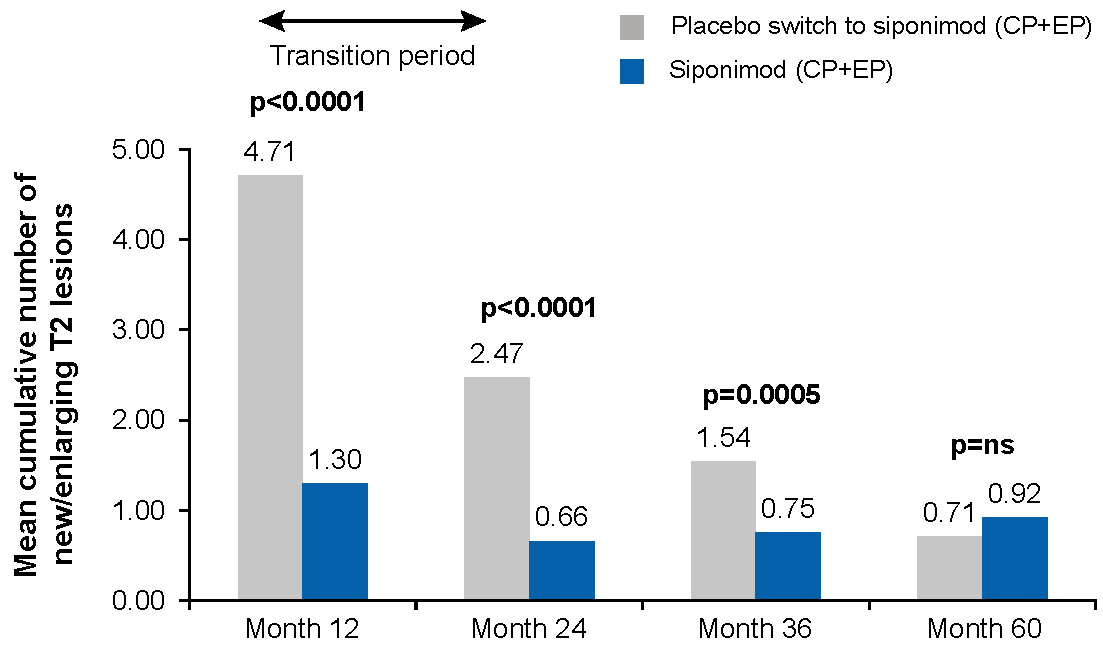
**

CP, core part; EP, extension part; ns, not significant.
