# Supplementary material for: Long-term efficacy and safety of siponimod in patients with secondary progressive multiple sclerosis: Analysis of EXPAND core and extension data up to >5 years
Source: Mult Scler. 2022 Apr 5;28(10):1591–605. doi: 10.1177/13524585221083194 (PMC9315196; doi:10.1177/13524585221083194)
Supplement: sj-docx-3-msj-10.1177_13524585221083194 – Supplemental material for Long-term efficacy and safety of siponimod in patients with secondary progressive multiple sclerosis: Analysis of EXPAND core and extension data up to >5 years [file sj-docx-3-msj-10.1177_13524585221083194.docx]

**Figure S3. Incidence of AEs per 100 PY in participants with active SPMS (IR ≥3 in the siponimod group during the core part^a^)**

**
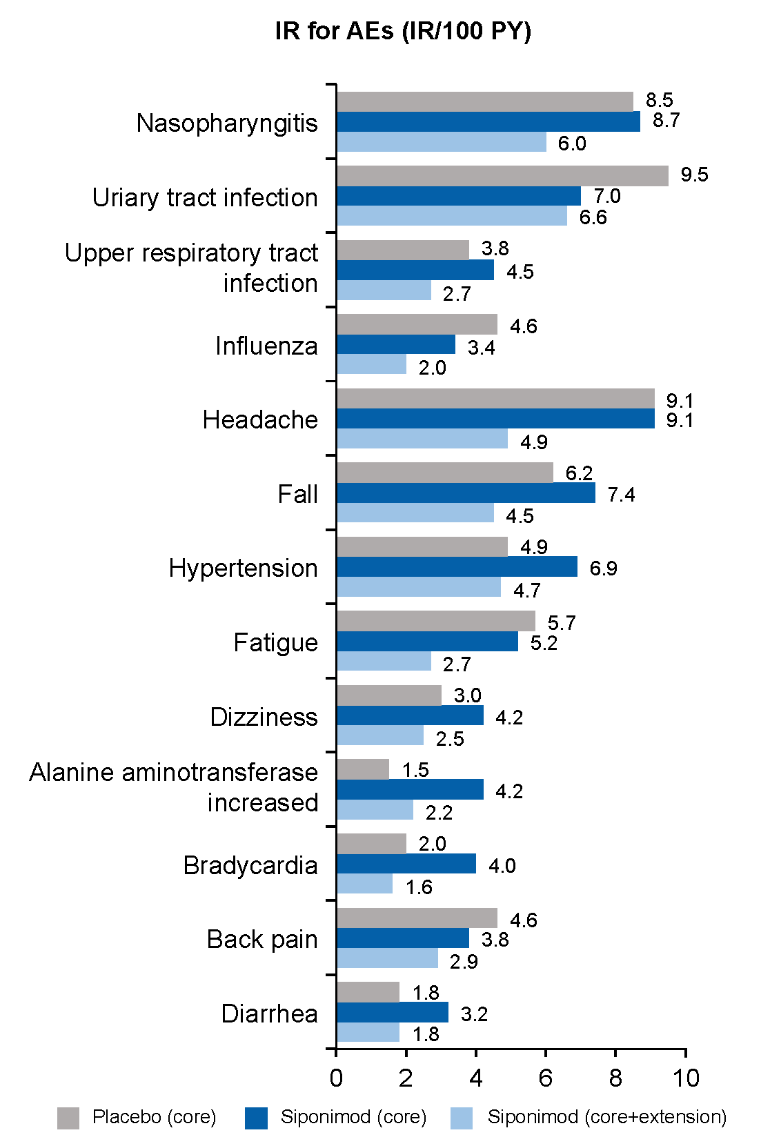
**

^a^IRs of the most common AEs reported in the core and extension parts were consistent with those of the core period.

AE, adverse event; IR, incidence rate; PY, patient-years; SPMS, secondary progressive multiple sclerosis.
