# Supplementary material for: Long-term efficacy and safety of siponimod in patients with secondary progressive multiple sclerosis: Analysis of EXPAND core and extension data up to >5 years
Source: Mult Scler. 2022 Apr 5;28(10):1591–605. doi: 10.1177/13524585221083194 (PMC9315196; doi:10.1177/13524585221083194)
Supplement: sj-docx-4-msj-10.1177_13524585221083194 – Supplemental material for Long-term efficacy and safety of siponimod in patients with secondary progressive multiple sclerosis: Analysis of EXPAND core and extension data up to >5 years [file sj-docx-4-msj-10.1177_13524585221083194.docx]

**Table S1. 6-month confirmed disability progression percentiles in the overall population of participants with SPMS**

| **Percentile (months)** | **Placebo-siponimod** | **Continuous siponimod** | **Delay (%)** |
| --- | --- | --- | --- |
| 25^th^ | 13.6 | 21.0 | 55 |
| 30^th^ | 18.4 | 29.8 | 62 |
| 35^th^ | 26.4 | 35.6 | 35 |
| 40^th^ | 33.9 | 44.9 | 32 |
| Median | 51.7 | Not reached | Not applicable |

SPMS, secondary progressive multiple sclerosis.
