# Supplementary material for: Long-term efficacy and safety of siponimod in patients with secondary progressive multiple sclerosis: Analysis of EXPAND core and extension data up to >5 years
Source: Mult Scler. 2022 Apr 5;28(10):1591–605. doi: 10.1177/13524585221083194 (PMC9315196; doi:10.1177/13524585221083194)
Supplement: sj-docx-5-msj-10.1177_13524585221083194 – Supplemental material for Long-term efficacy and safety of siponimod in patients with secondary progressive multiple sclerosis: Analysis of EXPAND core and extension data up to >5 years [file sj-docx-5-msj-10.1177_13524585221083194.docx]

**Table S2. 6-month confirmed disability progression percentiles in the populations of participants with active and non-active SPMS**

| **Percentile (months)** | **Placebo-siponimod** | **Continuous siponimod** |
| --- | --- | --- |
| **Active SPMS** | | |
| 25^th^ | 12.0 | 21.3 |
| 30^th^ | 15.2 | 28.1 |
| 40^th^ | 28.1 | 43.5 |
| Median | 48.0 | Not reached |
| **Non-active SPMS** | | |
| 25^th^ | 15.4 | 21.0 |
| 30^th^ | 21.5 | 31.5 |
| 40^th^ | 41.3 | 44.9 |

SPMS, secondary progressive multiple sclerosis.
