# Supplementary material for: Long-term efficacy and safety of siponimod in patients with secondary progressive multiple sclerosis: Analysis of EXPAND core and extension data up to >5 years
Source: Mult Scler. 2022 Apr 5;28(10):1591–605. doi: 10.1177/13524585221083194 (PMC9315196; doi:10.1177/13524585221083194)
Supplement: sj-docx-6-msj-10.1177_13524585221083194 – Supplemental material for Long-term efficacy and safety of siponimod in patients with secondary progressive multiple sclerosis: Analysis of EXPAND core and extension data up to >5 years [file sj-docx-6-msj-10.1177_13524585221083194.docx]

**Table S3. 6-month confirmed clinically meaningful worsening in CPS percentiles in the overall population of participants with SPMS**

| **Percentile (months)** | **Placebo-siponimod** | **Continuous siponimod** | **Delay (%)** |
| --- | --- | --- | --- |
| 25^th^ | 18.3 | 29.6 | 62 |
| 30^th^ | 26.4 | 39.0 | 48 |
| 35^th^ | 34.1 | 52.4 | 54 |
| 40^th^ | 41.3 | Not reached | Not applicable |

CPS, cognitive processing speed; SPMS, secondary progressive multiple sclerosis.
