# Supplementary material for: Long-term efficacy and safety of siponimod in patients with secondary progressive multiple sclerosis: Analysis of EXPAND core and extension data up to >5 years
Source: Mult Scler. 2022 Apr 5;28(10):1591–605. doi: 10.1177/13524585221083194 (PMC9315196; doi:10.1177/13524585221083194)
Supplement: sj-docx-7-msj-10.1177_13524585221083194 – Supplemental material for Long-term efficacy and safety of siponimod in patients with secondary progressive multiple sclerosis: Analysis of EXPAND core and extension data up to >5 years [file sj-docx-7-msj-10.1177_13524585221083194.docx]

**Table S4. 6-month confirmed clinically meaningful worsening in CPS percentiles in the populations of participants with active and non-active SPMS**

| **Percentile (months)** | **Placebo-siponimod** | **Continuous siponimod** |
| --- | --- | --- |
| **Active SPMS** | | |
| 25^th^ | 17.4 | 25.2 |
| 30^th^ | 18.7 | 37.6 |
| 40^th^ | 33.4 | Not reached |
| Median | 55.5 | Not reached |
| **Non-active SPMS** | | |
| 25^th^ | 26.0 | 30.4 |
| 30^th^ | 34.1 | 41.0 |
| 40^th^ | 48.5 | 64.9 |

CPS, cognitive processing speed; SPMS, secondary progressive multiple sclerosis.
