# Supplementary material for: Long-term efficacy and safety of siponimod in patients with secondary progressive multiple sclerosis: Analysis of EXPAND core and extension data up to >5 years
Source: Mult Scler. 2022 Apr 5;28(10):1591–605. doi: 10.1177/13524585221083194 (PMC9315196; doi:10.1177/13524585221083194)
Supplement: sj-docx-8-msj-10.1177_13524585221083194 – Supplemental material for Long-term efficacy and safety of siponimod in patients with secondary progressive multiple sclerosis: Analysis of EXPAND core and extension data up to >5 years [file sj-docx-8-msj-10.1177_13524585221083194.docx]

**Table S5. AEs of special interest (overall population)**

|  | **Core part** | | **Core+extension part** |
| --- | --- | --- | --- |
| **AEs** | **Placebo N=546; n (IR; per 100 PY)** | **Siponimod 2 mg  N=1099;  n (IR; per 100 PY)** | **Siponimod 2 mg N=1517;  n (IR; per 100 PY)** |
| Infections and infestations^a^ | 270 (50.2) | 545 (48.8) | 958 (37.3) |
| Varicella zoster virus infections^b^ | 4 (0.5) | 34 (2.0) | 86 (1.8) |
| Herpes simplex virus infections^b^ | 11 (1.3) | 25 (1.5) | 44 (0.9) |
| Bradyarrhythmia during dose titration^b^ | 63 (8.3) | 186 (12.6) | 297 (7.0) |
| Macular oedema^b^ | 1 (0.1) | 20 (1.2) | 31 (0.6) |
| Hypertension^c*^ | 51 (6.6) | 139 (8.9) | 252 (5.8) |
| Liver function tests elevated^*^ | 22 (2.7) | 146 (9.3) | 253 (5.7) |
| Suicidality^c^ | 4 (0.5) | 18 (1.0) | 25 (0.5) |
| Lymphopenia^b*^ | 0 (0) | 16 (0.9) | 175 (3.7)** |
| Malignancies^c^ | 14 (1.7) | 21 (1.2) | 78 (1.6) |
| Basal cell carcinoma^d^ | 7 (0.8) | 12 (0.7) | 47 (0.9) |

*Investigator-reported laboratory AEs.
****Absolute lymphocyte counts were blinded in the core part but not in the extension, leading to increased lymphopenia in the extension study
^a^System organ class; ^b^Novartis MedDRA query; ^c^Standard MedDRA query (MedDRA version 19.0); ^d^Preferred term.
IR exposure-adjusted incidence rate computed as number of participants with an AE divided by total exposure for the AE (i.e. cumulative exposure until first occurrence or until end of follow-up

AEs, adverse events; IR, incidence rate; MedDRA, Medical Dictionary for Regulatory Activities.
