# Supplementary material for: Long-term efficacy and safety of siponimod in patients with secondary progressive multiple sclerosis: Analysis of EXPAND core and extension data up to >5 years
Source: Mult Scler. 2022 Apr 5;28(10):1591–605. doi: 10.1177/13524585221083194 (PMC9315196; doi:10.1177/13524585221083194)
Supplement: sj-pdf-9-msj-10.1177_13524585221083194 – Supplemental material for Long-term efficacy and safety of siponimod in patients with secondary progressive multiple sclerosis: Analysis of EXPAND core and extension data up to >5 years [file sj-pdf-9-msj-10.1177_13524585221083194.pdf]

# MS Journal Appendix for MRI methodology

| Hardware | |
|---|---|
| Field strength | 1.5 Tesla or 3.0 Tesla |
| Manufacturer | Various scanners used by NeuroRX, Montreal, Canada, H2X 3P9 |
| Model | Various software used by NeuroRX, Montreal, Canada, H2X 3P9 |
| Coil type<br>(e.g. head, surface) | |
| Number of coil channels | |

| Acquisition sequence | | |
|---|---|---|
| Type<br>(e.g. FLAIR, DIR, DTI, fMRI) | PD-weighted, T2-weighted, FLAIR | |
| Acquisition time | | |
| Orientation | True mid-line sagittal | |
| Alignment<br>(e.g. anterior commissure/poster commissure line) | Indicated lesions were checked for correspondence to a lesion on at least one of the appropriate images, and it was ensured that the lesion was completely within tissue | |
| Voxel size | | |
| TR | | |
| TE | | |
| TI | | |
| Flip angle | | |
| NEX | | |
| Field of view | | |
| Matrix size | | |
| Parallel imaging | | No |
| If used, parallel imaging method:<br>(e.g. SENSE, GRAPPA) | | |
| Cardiac gating | | No |
| If used, cardiac gating method:<br>(e.g. PPU or ECG) | | |
| Contrast enhancement | | No |

| Acquisition sequence | |
|---|---|
| If used, provide name of contrast agent, dose and timing of scan post-contrast administration | N/A |
| Other parameters: | N/A |

| Image analysis methods and outputs | |
|---|---|
| ***Lesions*** | |
| Type<br>(e.g. Gd-enhancing, T2-hyperintense, T1-hypointense) | T2 new/englarging, T2 lesion volume change |
| Analysis method | An initial segmentation of the scan was carried out based on a set of starting labels provided by software that employs a Bayesian classifier with anatomical prior knowledge |
| Analysis software | |
| Output measure<br>(e.g. count or volume [ml]) | |
| ***Tissue volumes*** | |
| Type<br>(e.g. whole brain, grey matter, white matter, spinal cord) | Total brain, cortical grey matter, thalamic volume |
| Analysis method | |
| Analysis software | JacobianAtrophy software (Nakamura et al., 2013) |
| Output measure<br>(e.g. absolute tissue volume in ml, tissue volume as a fraction of intracranial volume, percentage change in tissue volumes) | |
| ***Tissue measures (e.g. MTR, DTI, T1-RT, T2-RT, T2*, T2', $^{1}$H-MRS, perfusion, Na)*** | |
| Type<br>(e.g. whole brain, grey matter, white matter, spinal cord, normal-appearing grey matter or white matter) | N/A |
| Analysis method | |
| Analysis software | |
| Output measure | |
| ***Other MRI measures (e.g. functional MRI)*** | |
| Type<br>(e.g. whole brain, grey matter, white matter, spinal cord, normal-appearing grey matter or white matter) | N/A |
| Analysis method | |
| Analysis software | |
| Output measure | |

**Other analysis details:**
